# Fabricating High Strength Bio-Based Dynamic Networks from Epoxidized Soybean Oil and Poly(Butylene Adipate-*co*-Terephthalate)

**DOI:** 10.3390/polym16162280

**Published:** 2024-08-11

**Authors:** Bin Xu, Zhong-Ming Xia, Rui Zhan, Ke-Ke Yang

**Affiliations:** The Collaborative Innovation Center for Eco-Friendly and Fire-Safety Polymeric Materials (MoE), National Engineering Laboratory of Eco-Friendly Polymeric Materials (Sichuan), College of Chemistry, Sichuan University, Chengdu 610064, China; xvbin1028@126.com (B.X.); 15928876074@163.com (Z.-M.X.); m19942331358@163.com (R.Z.)

**Keywords:** epoxidized soybean oil, dynamic networks, poly(butylene adipate-co-terephthalate), thiourethane

## Abstract

Amid the rapid development of modern society, the widespread use of plastic products has led to significant environmental issues, including the accumulation of non-degradable waste and extensive consumption of non-renewable resources. Developing healable, recyclable, bio-based materials from abundant renewable resources using diverse dynamic interactions attracts increasing global attention. However, achieving a good balance between the self-healing capacity and mechanical performance, such as strength and toughness, remains challenging. In our study, we address this challenge by developing a new type of dynamic network from epoxidized soybean oil (ESO) and poly(butylene adipate-*co*-terephthalate) (PBAT) with good strength and toughness. For the synthetic strategy, a thiol–epoxy click reaction was conducted to functionalize ESO with thiol and hydroxyl groups. Subsequently, a curing reaction with isocyanates generated dynamic thiourethane and urethane bonds with different bonding energies in the dynamic networks to reach a trade-off between dynamic features and mechanical properties; amongst these, the thiourethane bonds with a lower bonding energy provide good dynamic features, while the urethane bonds with a higher bonding energy ensure good mechanical properties. The incorporation of flexible PBAT segments to form the rational multi-phase structure with crystalline domains further enhanced the products. A typical sample, OTSO_100_-PBAT_100_, exhibited a tensile strength of 33.2 MPa and an elongation at break of 1238%, demonstrating good healing capacity and desirable mechanical performance. This study provides a promising solution to contemporary environmental and energy challenges by developing materials that combine mechanical and repair properties. It addresses the specific gap of achieving a trade-off between tensile strength and elongation at break in bio-based self-healing materials, promising a wide range of applications.

## 1. Introduction

Plastic products are extensively used across various industries such as packaging, construction, medical, electronics, and toys due to their light weight, durability, affordability, and ease of processing; however, their widespread use results in substantial waste generation and emissions. This plastic waste has escalated into a significant environmental issue, posing a dual challenge: ecological damage and the consumption of non-renewable resources [1,2,3]. The unsustainable pattern of resource consumption threatens the long-term health and well-being of our planet. It is crucial to reduce reliance on non-renewable resources and promote sustainable practices to protect our environment for future generations. 

To address these challenges, researchers worldwide have proposed various solutions, including developing bio-based materials from renewable resources, biodegradable plastics for disposable products, self-healing polymers (SHPs) for durable products, and recycling and upcycling waste [4,5,6,7,8,9]. Among these, SHPs stand out due to their remarkable ability to repair themselves automatically under suitable conditions—such as heat [10], pH change [11], pressure [12], radiation [13], or magnetic field [14]—when they are damaged or undergo wear and tear [15,16,17]. Based on their healing mechanism, SHPs can be categorized into extrinsic and intrinsic types. Extrinsic SHPs rely on external reagents and their repair ability diminishes when the reagents are exhausted [18,19]. In contrast, intrinsic SHMs are healed by their inherent dynamic interactions, such as supramolecular interactions (e.g., hydrogen bond [20], π-π interaction [21], and metal–ligand coordination [22]), and dynamic covalent bonds (e.g., cycloaddition [23], Schiff base [24], acylhydrazone [25], disulfide [26], Michael addition [27], transesterification [28], urethane [29], and thiourethane [30]). 

Traditional thermosets generate significant plastic waste due to crosslink networks, which cannot be healed, reprocessed, and recycled after the service periods [31,32,33,34]. To address this issue, covalent adaptable networks (CANs) [35,36]—first proposed by Bowman et al. in 2005—offer a solution with dynamic covalent bonds based on associative or dissociative exchange mechanisms, allowing reprocessing and recycling. In 2011, Leibler et al. [37] introduced the concept of “vitrimer,” which refers to a thermosetting resin with an associative exchange feature. Amongst various dynamic bonds, transesterification, urethane, and thiourethane are typical associative exchange types. Sulfur-containing dynamic bonds attract much attention from researchers due to the relatively low bond energy compared to oxygen- or nitrogen-containing bonds, resulting in better dynamic characteristics [30]. Thiourethane, formed by thiol-isocyanate, has a lower bond energy than urethane, providing better dynamic features in mild conditions.

While dynamic bonds are crucial for a vitirmer, the resources of the materials are also important. From a sustainability perspective, developing bio-based vitrimers from renewable resources is promising [38,39]. Plant oils, which can be obtained in large quantities from agricultural products, have natural unsaturated bonds suitable for chemical modification. Various studies have explored plant oils as alternatives for petroleum-based polymers [40,41,42,43,44]. For instance, epoxidized soybean oil (ESO) is a popular raw material for designing bio-based vitrimers due to its abundant reactive epoxy groups. Song et al. [45] developed a system using epoxidized soybean oil and vanillin to create a network based on the dynamic three-dimensional hydrogen bonds and dynamic imine linkages. Similarly, Zhao et al. [46] utilized Schiff bases derived from vanillin with 1,2-dimethylimidazole to cure ESO. However, achieving a trade-off between self-healing capacity and mechanical performance, such as strength and toughness, remains challenging. 

In our study, we aim to develop a new ESO-based dynamic network with good strength and toughness by incorporating thiourethane and urethane bonds together through a meticulous synthetic strategy and rational multi-phase structure with crystalline domains. By using the thiol–epoxy click reaction [47], ESO can be efficiently thiol-functionalized to OTSO and also generate hydroxyl groups simultaneously via the ring-opening of the epoxy group. This enables the formation of thiourethane and urethane bonds in dynamic networks during the curing process by adding isocyanates. The two types of dynamic bonds have very similar structures but different bonding energies, thiourethane bonds with a lower bonding energy provide good dynamic features, while urethane bonds with a higher bonding energy ensure good mechanical properties. To balance the strength and toughness, we also incorporated a biodegradable, flexible, and crystalline segment of poly(butyleneadipate-*co*-terephthalate) (PBAT) into the networks to produce OTSO-PBAT networks to form a rational multi-phase structure. The composition of OTSO-PBAT was optimized, and a typical sample, OTSO_100_-PBAT_100_, with good healing capacity and desirable mechanical performance (tensile strength of 33.2 MPa and elongation at break of 1238%) was achieved. The originality of this research lies in developing a dynamic network that balances mechanical properties and self-healing capabilities using ESO and PBAT. This addresses the specific gap of achieving a trade-off between tensile strength and elongation at break in bio-based self-healing materials. It provides a promising solution to contemporary environmental and energy challenges by developing materials that combine mechanical and repair properties. The high adaptability of these dynamic bonds with the structure of renewable resources will be a good reference for future research to develop vitrimers from other renewable resources.

## 2. Materials and Methods

### 2.1. Materials 

Epoxidized soybean oil (ESO, AR grade, Mn = 950 g·mol^−1^), Isophorone diisocyanate (IPDI, 98%), Dibutyltin dilaurate (DBTDL, 95%), 1,5- Diazabicyclo [4.3.0] non-5-ene (DBN, 98%), 1,3-Propanedithiol (PDT, 98%), and 1,3-Propanediol (PDO, 98%) were purchased from Aladdin reagent, Shanghai, China. 3,6-Dioxa-1,8-octanedithiol (DODT, 98%) was obtained from TCI (Shanghai) Development Corp., Shanghai, China. 1,2-Dichloroethane (DCE, 99.5%, extra dry with molecular sieves, water ≤ 50 ppm) was purchased from Innochem, Beijing, China. Dichloromethane (DCM, AR grade) and Triethanolamine (TEA, 98%) were purchased from Kelong Reagent Corp., Chengdu, China. All materials and reagents described herein were used without further purification. Poly(butyleneadipate-*co*-terephthalate) (PBAT) with a molecular weight of 9000 g/mol was obtained from Red Avenue New Material Group Corp., Shanghai, China, and dried in an electric blast oven at 80 °C for 12 h before use.

### 2.2. Synthesis of Octane Thiol Soybean Oil (OTSO)

**Figure 1:**
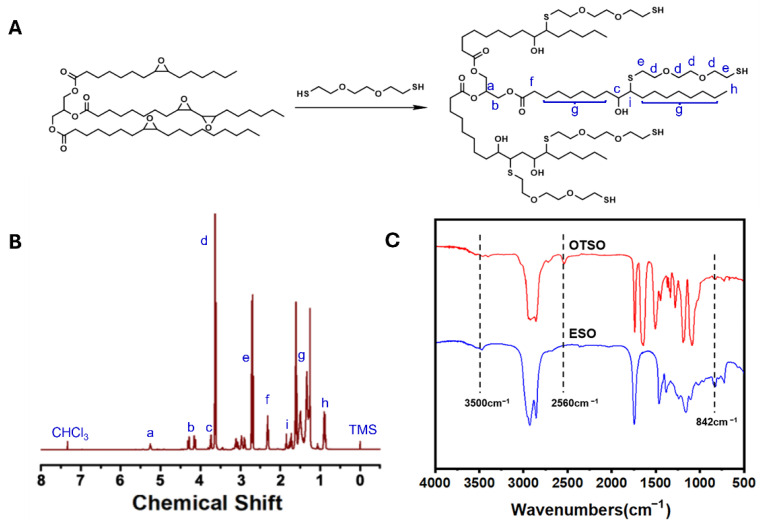
(**A**) Synthetic route, (**B**) 1H NMR spectrum, and (**C**) FT-IR spectrum of OTSO.

The synthesis route of OTSO is illustrated in Figure 1A. Initially, the reaction solvent dichloromethane (DCM, 120 mL) was added to a 250 mL round-bottom flask, followed by 3,6-dioxaoctane-1,8-dithiol (DODT; 17.09 mL, 105 mmol) and the catalyst triethylamine (TEA, 0.73 mL, 5.25 mmol). The mixture was stirred until dissolved; then, the reaction material of 23.82 mL (25 mmol) ESO was added. The reaction mixture was then reacted at room temperature with stirring for 3 h to yield a colorless and transparent solution. The product was subjected to rotary evaporation at 40 °C to remove solvent, resulting in a colorless, transparent oil with low viscosity.

### 2.3. Synthesis of the OTSO-IPDI Network (Control Group)

**Figure 2:**
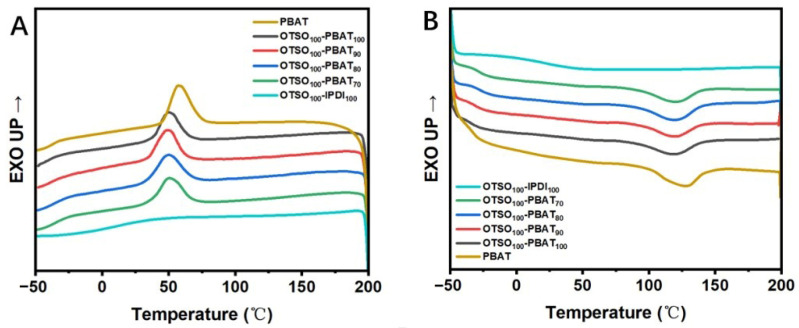
DSC curves of OTSO−based networks and PBAT: (**A**) cooling scan and (**B**) heating scan with a scanning rate of 10 °C/min.

As depicted in the synthesis route (Figure 2), OTSO (5 mL, 5 mmol) was added to a 50 mL round-bottom flask equipped with a septum and cooled in an ice bath. Anhydrous DCM (25 mL) was added under stirring, followed by the crosslinking agent IPDI (1.05 mL, 5 mmol) and the catalyst DBN (0.06 mL, 0.05 mmol). After stirring to ensure uniform mixing, the reaction mixture was rapidly poured into a Teflon dish for casting for 24 h. After evaporation of the solvent, the casting film was further crosslinked by placing it into a 120 °C oven for 8 h. The resulting film was vacuum-dried to constant weight.

### 2.4. Synthesis of OTSO-PBAT Network

By adjusting the feed ratio between OTSO and PBAT, a series of OTSO*_x_*-PBAT*_y_* were synthesized (Figure 2). Here, the subscript “*x*” and “*y*” represent the molar ratio of OTSO to NCO-terminated PBAT, summarized in Table S1. Taking OTSO_100_-PBAT_100_ as an example, the synthetic route is as follows: pre-dried PBAT (5 g) was added to a flask with a septum and vacuumed for 6 h to remove free moisture before purging with nitrogen. Anhydrous 1,2-dichloroethane was added, and the solution was stirred until dissolved. A 2.4 molar excess of IPDI and 1.5 mol% catalyst DBTDL were added and the end-capping reaction was conducted at 75 °C for 3 h. OTSO (0.8 g) was then added to react with the hydroxyl groups in the chain segments. After reacting for 6 h, when the system became a weak gel and the viscosity increased, a 1.25-fold molar excess of IPDI and the catalyst DBN relative to OTSO were added to start the reaction between the NCO groups and thiol groups in OTSO. After thorough stirring, the reaction mixture was rapidly poured into a Teflon dish for casting for 24 h, and after the solvent was evaporated, the film was put into an oven at 120 °C for 8 h.

### 2.5. Characterization

#### 2.5.1. Nuclear Magnetic Resonance Hydrogen Spectrum (^1^H-NMR)

The structural characterization of the compounds was performed using a Bruker AV400 400MHz NMR spectrometer, Billerica, MA, USA., with deuterated chloroform (CDCl_3_) as the solvent and tetramethylsilane (TMS) as the internal standard.

#### 2.5.2. Fourier-Transform Infrared Spectroscopy (FT-IR)

FT-IR spectra were obtained using a Nicolet 6700 Fourier-Transform Infrared Spectrometer, Waltham, MA, USA., in the wavenumber range of 600–4000 cm^−1^ using transmission mode.

#### 2.5.3. Gel Swelling Test

Samples were cut into small pieces and their original mass (*m*_0_) was recorded. The samples were then soaked in dichloromethane for 24 h. After wiping off the surface solvent, the swollen samples were immediately weighed (*m*_1_), followed by drying the swollen samples to constant weight (*m*_2_). The gel content (*G*) and swelling rate (*S*) were calculated using the formulas in Equation (1) and Equation (2), respectively.

(1) $$G=\frac{m_{2}}{m_{0}}\times 100\%$$

(2) $$S=\frac{m_{1}}{m_{2}}\times 100\%$$

#### 2.5.4. Differential Scanning Calorimetry Test (DSC)

The thermal properties of the material were analyzed using a DSC-Q200 analyzer from TA Instruments, New Castle, DE, USA. Initially, the material was heated to 200 °C and maintained for 2 min to eliminate thermal history. Subsequently, it was cooled to −50 °C and then heated to 200 °C at a scanning rate of 2 °C/min.

#### 2.5.5. Thermal Relaxation Test

The thermal relaxation behavior of the materials was tested using a TA Instruments DMA Q800 dynamic thermal mechanical analyzer, New Castle, DE, USA. Samples measuring 30 mm × 5 mm × 1 mm were first heated to 80 °C and stabilized for 5 min, then stretched to 10% deformation and held for 60 min to record the thermal relaxation behavior.

#### 2.5.6. Static Tensile Test

The mechanical properties of the samples were tested using an Instron Model 3366 universal testing machine, Norwood, MA, USA., equipped with a 1 KN sensor. Samples were cut into dumbbell shapes with a gauge length of 20 mm, a thickness of 0.7 mm, and a width of 4 mm. The stretching rate was set to 50 mm/min, and each sample was tested at least three times for averaging.

#### 2.5.7. Self-Healing Performance Test

The self-healing performance of the materials was observed using a Nikon ECLIPSE LV100POL, Tokyo, Japan., polarized light microscope and thermal table (HSC621V) in the non-polarized light mode. Digital photographs of the material surface were recorded at certain temperatures and different time intervals using a digital camera. 

## 3. Results and Discussion

### 3.1. Preparation of Thiol-Terminated ESO (OTSO)

Before the reaction, the ESO was titrated using the hydrochloric acid–acetone method, indicating that each ESO molecule contained an average of 3.5 epoxy groups. Then, OTSO was synthesized through a thiol–epoxy click reaction between ESO and DODT (Figure 1A). The resulting product was characterized by ^1^H-NMR with CDCl_3_ as the solvent, as illustrated in Figure 1B. In the ^1^H-NMR spectrum, peaks at 5.28 ppm (δHa) and 4.26 ppm (δHb) were attributed to the tertiary and secondary hydrogens in the glycerol structure of soybean oil, with a peak area ratio of 1:4, respectively. The peak at 3.75 ppm (δHc) corresponded to the tertiary hydrogen linked to the hydroxyl group formed by the ring-opening reaction. Peaks at 3.62 ppm (δHd) and 2.74 ppm (δHe) were associated with the hydrogen on the carbon chain of DODT added by the click reaction. Peaks at 2.42 ppm (δHf), 1.2–1.8 ppm (δHg), and 0.91 ppm (δHh) corresponded to the hydrogens on the fatty acid carbon chain of soybean oil. The peak at 1.85 ppm (δHi) was attributed to the tertiary hydrogen added to the fatty acid carbon chain by the thiol group. The residual solvent peak of CDCl_3_ appeared at 7.26 ppm. Based on the peak area ratio of δHa to δHi as 1:2.5, it was inferred that each ESO molecule on average possesses 2.5 –SH groups. FT-IR characterization of the product was also conducted, as shown in Figure 1C. Compared with ESO, the appearance of the absorption peak at 2560 cm^−1^ corresponded to the -SH groups linked to ESO molecules, and a very broad absorption peak at 3500 cm^−1^ corresponded to the hydroxyl groups formed from the ring-opening of the epoxy group during the thiol–epoxy click reaction. Both ^1^H-NMR and FT-IR confirmed that thiol groups were successfully introduced into ESO molecules, with an average of 2.5 –SH groups per ESO molecule. 

### 3.2. Curing and Structural Characterization of the OTSO-PBAT Network

OTSO*_x_*-PBAT*_y_* networks were prepared through the curing reaction between the mixture of OTSO, PBAT, and IPDI. Before the preparation, to verify whether –OH groups would compete with –SH groups in curing, a validation model was established: a mixture of 1,3-Propanedithiol (PDT) and 1,3-Propanediol (PDO) was reacted with IPDI under different catalysts and characterized by FT-IR, as shown in Figure S1. The absorption peak near 3300 cm^−1^ corresponds to the N–H bond formed by the addition of -NCO groups and the absorption peak at 1700 cm^−1^ corresponds to the C=O bond of urethane and thiourethane links within the molecule. Under the catalysis of DBN, the absorption peak for N–H in the mixed product shifts to a lower wavenumber than that of under DBTDL, and the absorption peak for C=O also shifts to a lower wavenumber. This shift is attributed to sulfur having a lower electronegativity compared to oxygen, resulting in a weaker electron-withdrawing effect and thus a redshift in the FT-IR spectrum. These results indicate the reactivity of –OH and –SH with isocyanates varies under different catalysts due to different reaction mechanisms. The reaction of –OH with isocyanates is catalyzed by coordination with organotin, while –SH groups add to isocyanates in the presence of organic bases. Thus, by controlling different reaction conditions, –OH and –SH can individually react with IPDI to form dynamic bonds, thereby enhancing mechanical properties while preserving their self-healing capabilities.

Then, the curing reactions of OTSO*_x_*-PBAT*_y_* were carried out under conditions of the sequential addition of DBTDL and DBN, as illustrated in Figure S2. A control sample of OTSO_100_-IPDI_100_ was also prepared without adding PBAT. Due to varying proportions of PBAT, different linking modes were formed within the network. Generally, in groups with a higher proportion of PBAT, most of the –OH on OTSO connects with PBAT, resulting in the polymer network’s stress-bearing parts being the less dynamic urethane bonds. Conversely, in groups with a lower proportion of PBAT, some –OH that did not react with PBAT could be linked with other OTSO molecules by excess IPDI, distributing the stress over parts of OTSO. Since the bond energy of the thiourethane bonds formed on OTSO molecules is lower than that of urethane bonds, they are more prone to breakage. With the addition of excess IPDI in the second step of the reaction, the quantity of –NCO groups far exceeded the quantity of –OH and –SH groups, assuming that all –OH and –SH participated in the reaction. 

### 3.3. Thermal Behaviors of the OTSO-PBAT Network

To study the curing process, differential scanning calorimetry (DSC) was employed to determine phase transitions of the mixture of OTSO, NCO-terminated PBAT, and IPDI and the reaction peaks within a temperature range of −50 to 150 °C at a heating rate of 5 °C/min. As shown in Figure S3, the melting point of ESO was observed at −25 °C, exhibiting a broad endothermic peak. A broad endothermic peak at 130 °C was observed, indicating the occurrence of a crosslinking reaction and formation of the network between OTSO and NCO-terminated PBAT. Notably, this broad endothermic peak also included the endothermic peak produced by the melting of PBAT. Subsequent DSC testing of PBAT indicated a melting temperature of 127 °C (Figure 2). Since the endothermic peak of the reactant is much broader than the melting endothermic peak of PBAT, it can be concluded that the reaction proceeded smoothly at this temperature. It is worth noting that at higher temperatures (>120 °C), although the curing rate would significantly increase, the resulting product might exhibit yellowing, potentially due to the oxidation of thiol groups in OTSO. Therefore, a reaction temperature of 120 °C was chosen for the subsequent reactions.

To investigate the degree of reaction and crosslinking density, swelling tests were conducted to obtain *G* and *S* values, as listed in Table S1. It was observed that with the increasing proportion of PBAT, the *G* values decreased from 90.1% of OTSO_100_-PBAT_70_ to 84.2% of OTSO_100_-PBAT_100_. This may be attributed to the long polymer chains of PBAT reducing the reactive probability of the terminal NCO groups. Conversely, the control group OTSO_100_-IPDI_100_ without NCO-terminated PBAT exhibited the highest *G* value of about 96.3%. The *S* value reflects the crosslinking density of the network. As expected, *S* increased with increasing PBAT proportions, indicating a decrease in the crosslinking density because the PBAT chains are significantly longer than the fatty acid chains in OTSO. Predictably, the control sample OTSO_100_-IPDI_100_ without PBAT exhibited the highest crosslinking density.

### 3.4. Dynamic Features of the OTSO-PBAT Network

DSC analyses were conducted to investigate the thermal behavior of the OTSO-PBAT networks, as shown in Figure 2, with corresponding data presented in Table 1. The OTSO_100_-IPDI_100_ sample, which has the highest crosslinking density, exhibits the highest *T*_g_ at −16 °C, indicating reduced mobility of its short-chain segments. No crystalline behavior was observed during the whole scanning process. As the crosslinking density decreases, the *T*_g_ correspondingly decreases, suggesting an increase in the mobility of the chain segments. Compared to pure PBAT, all OTSO-PBAT samples exhibited lower melting temperatures (*T*_m_), enthalpy of melting (Δ*H*_m_), crystallization temperatures (*T*_c_), and enthalpy of crystallization (Δ*H*_c_). This indicates that the crosslinking and introduction of OTSO reduces the overall crystallinity of the network. Within this series, as the proportion of PBAT increases, both Δ*H*_m_ and Δ*H*_c_ show a very slight increase. This can be attributed to the lower crosslinking density, which makes the polymer more prone to crystallization. The existence of the crystalline domains of PBAT segments indicates that the networks exhibit a multi-phase structure that may contribute to enhancing the OTSO-PBAT samples.

### 3.5. Mechanical Properties of the OTSO-PBAT Network

**Figure 3:**
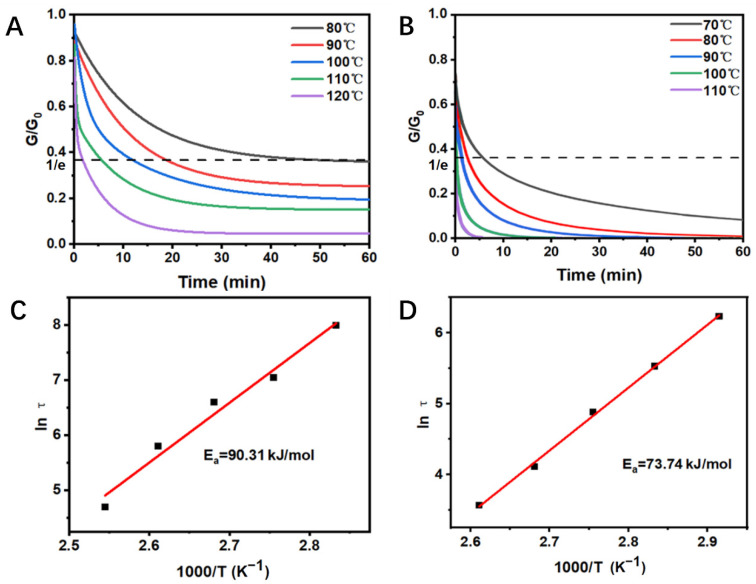
Stress relaxation curves of (**A**) OTSO_100_-PBAT_100_ and (**B**) TSO_100_-IPDI_100_, and their apparent activation energy (**C**,**D**), respectively.

To investigate the dynamic feature of the OTSO-PBAT network containing thiourethane bonds, stress relaxation experiments were conducted on OTSO100-PBAT100 (Figure 3A) and TSO_100_-IPDI_100_ networks (Figure 3B). The samples were stretched to 10% strain at different temperatures by using a DMA. Figure 3A,B display the changes in the relaxation modulus at temperatures ranging from 80 to 120 °C, where the relaxation time (*τ*^∗^) is defined as the moment when the material relaxes to 1/e of its initial modulus (*G*/*G*_0_ = 1/e). At 90 °C, the OTSO_100_-PBAT_100_ sample relaxed to below 40% of its residual stress within 20 min due to the exchange of thiourethane bonds. At 120 °C, the sample relaxed to below 40% strain within 2 min as the PBAT chains reached their melting temperature, increasing the polymer chains’ mobility and allowing the rapid rearrangement of the topological structure, leading to quick stress relaxation. Notably, even after relaxing for 60 min at 120 °C, there was still 5% residual stress, attributed to the higher exchange temperature of urethane bonds in the network. However, when the temperature was raised to 130 °C, the urethane bonds also began to exchange rapidly, completely disrupting the network structure, and the stress was fully released, making the data at 130 °C not valuable for reference. Through detailed analysis of the relaxation process, it was found that the system’s relaxation follows the Maxwell model, described by the Arrhenius Equation (Equation (3)).

*τ*^∗^ = τ_0_exp(*E*_a_/R*T*) (3)

where τ^∗^ is the time required to reach a specific *G*/*G*_0_ value (1/e) at a given temperature; *E*_a_ is the apparent activation energy of bond exchange; *T* is the experimental temperature; and R is the molar gas constant (8.314 J·K^−1^·mol^−1^). The *E*_a_ of OTSO_100_-PBAT_100_ was calculated to be 90.31 kJ·mol^−1^ with a correlation coefficient of 0.9769 (Figure 3C), while that of TSO_100_-IPDI_100_ was 73.74 kJ·mol^−1^ with a correlation coefficient of 0.9919 (Figure 3D). This may be attributed to the OTSO-PBAT series having lower dynamic bond densities while the PBAT with long chains was incorporated into the networks. 

**Figure 4:**
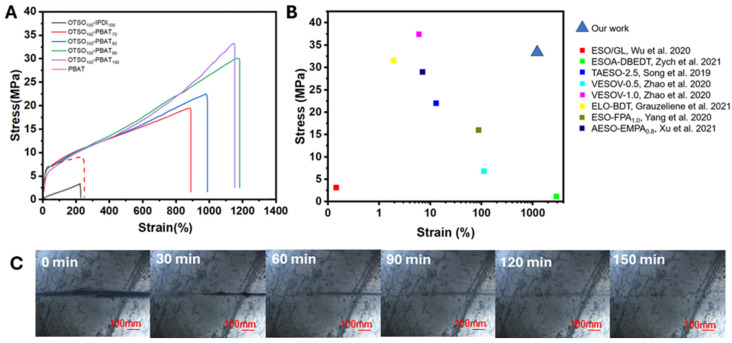
(**A**) Stress–strain curves of samples. (**B**) Comparison of mechanical properties of this work with relevant references as noted. [42,45,46,48,49,50,51] (**C**) Self-healing processes of OTSO_100_-PBAT_100_ at 120 °C within 2.5 h.

The mechanical properties of the OTSO-PBAT networks were investigated using a universal testing machine, with the stress–strain curves presented in Figure 4A and the relevant data summarized in Table 2. The OTSO-PBAT samples exhibited characteristics of hard elastic materials, while the control sample OTSO-IPDI displayed typical elastomeric behavior. This hard elasticity is attributed to the crystalline phase of PBAT. Compared to the OTSO-IPDI—with a tensile strength of 3.5 MPa and elongation at break of 155%—the mechanical performance of the OTSO-PBAT series significantly increased with the increase in PBAT content. Particularly, OTSO_100_-PBAT_100_ exhibits a tensile strength of 33.2 MPa and an elongation at break of 1238%, representing 949% and 798% increases over the control group, respectively. Neat PBAT with low molecular weight, in contrast, exhibited a tensile strength of 8.2 MPa and an elongation at break of 280%. 

Introducing flexible and crystalline PBAT segments can dissipate energy during stretching. Additionally, the connection between PBAT and OTSO involves more stable urethane bonds, while the links in the OTSO-IPDI control sample involve more active thiourethane bonds with lower bonding energy. The formation of the crosslinking structure also further strengthens the material’s tensile resistance. It can be inferred from the tensile curves that as the proportion of PBAT increases, the tensile resistance of the crosslinked network correspondingly improves. More crystalline regions provided by additional PBAT offer stronger stress dissipation capabilities to the crosslinked network. Fewer connections between OTSO molecules via urethane bonds mean that stress is primarily borne by PBAT chains. The longer chains of PBAT provide more entanglement for dissipating stress, whereas the shorter chains of OTSO have less entanglement and quickly reach their limit of extension under stress, leading to direct network rupture. A comparison of mechanical performance of ESO vitrimers between our work and the relevant reported works is illustrated in Figure 4B. It clearly shows that our approach with ESO and PBAT incorporates thiourethane and urethane bonds, achieving a superior balance between tensile strength and elongation at break.

### 3.6. Self-Healing Capacity of the OTSO-PBAT Network

Due to the structural similarities between urethane and thiourethane bonds, exchange reactions can occur during the repair process, thereby ensuring relatively good self-healing performance. The self-healing capability of OTSO_100_-PBAT_100_ was finally evaluated by scratching the surface of a sample with a blade and then putting it onto a hot stage at 120 °C under a polarizing microscope, as illustrated in Figure 4C. It was observed that the scratch on the sample essentially healed after 2.5 h at 120 °C, demonstrating its desirable repair ability.

## 4. Conclusions

In summary, we successfully fabricated a new type of bio-based OTSO-PBAT dynamic network via a two-step approach. Initially, a first thiol–epoxy click reaction was performed on ESO to obtain OTSO functionalized with thiol and hydroxyl groups. This was followed by a co-curing reaction with flexible and crystalline PBAT using isocyanate as a hardener to produce a series of target OTSO-PBAT networks with desirable mechanical performance and a self-healing capacity. The OTSO precursor and the final networks were characterized using ^1^H-NMR, FT-IR, and swelling tests. The thermal properties of the OTSO-PBAT networks were evaluated by DSC, revealing a *T*_g_ range from −16 to −23 °C, with different feed ratios between OTSO and PBAT. The formation of the crosslinked structure was found to slightly suppress the crystallization ability of PBAT. The dynamic feature of the OTSO-PBAT network was demonstrated by stress relaxation experiments, revealing an apparent activation energy of dynamic bonds of 90.31 kJ·mol^−1^. Mechanical performance tests further validated that the incorporation of PBAT into the polymer network enhanced the tensile strength and elongation at break of materials. The OTSO_100_-PBAT_100_ sample achieved an elongation at break of 1238% and a tensile strength of 33.2 MPa, marking a 949% increase in tensile strength and a 798% increase in elongation at break compared to the control group without PBAT. The self-healing performance of the damaged sample was observed by POM, demonstrating rapid self-healing at 120 °C. 

## Figures and Tables

**Table 1:** The relevant thermal data of OTSO-based networks recorded from DSC curves.

Sample	*T*_g_ (°C)	*T*_m_ (°C)	Δ*H*_m_ (J/g)	*T*_c_ (°C)	Δ*H*_c_ (J/g)
PBAT	−20	127.23	16.08	57.03	22.33
OTSO_100_-IPDI_100_	−16	-	-	-	-
OTSO_100_-PBAT_70_	−20	120.31	11.49	49.23	16.11
OTSO_100_-PBAT_80_	−23	119.23	11.64	50.38	16.08
OTSO_100_-PBAT_90_	−22	120.01	12.26	50.51	16.37
OTSO_100_-PBAT_100_	−23	120.38	12.42	50.81	17.70

**Table 2:** The mechanical properties of PBAT and OTSO-based networks recorded from tensile tests.

Sample	Strain at Break (%)	Stress (MPa)
PBAT	246 ± 9	8.0 ± 0.23
OTSO_100_-IPDI_100_	248 ± 169	4.1 ± 0.2
OTSO_100_-PBAT_70_	887 ± 139	17.4 ± 2.5
OTSO_100_-PBAT _80_	1014 ± 22	22.1 ± 3.8
OTSO_100_-PBAT _90_	1176 ± 37	30.6 ± 2.8
OTSO_100_-PBAT _100_	1239 ± 19	33.2 ± 1.9

## Data Availability

Data are contained within the article and Supplementary Materials. And further inquiries can be directed to the corresponding author. [insert reason here].

## Supplementary Materials

The following supporting information can be downloaded at: https://www.mdpi.com/article/10.3390/polym16162280/s1, Figure S1: (A) The reaction of IPDI with a mixture of model molecules1,3-Propanedithiol (PDT) and 1,3-Propanediol (PDO) under different catalysts; (B) FT-IR spectra of resultant products; Figure S2: Synthetic strategy of OTSO-IPDI and OTSO-PBAT Networks; Figure S3: DSC curve of OTSO, NCO-terminated PBAT and IPDI within a temperature range of −50 to 150 °C at a heating rate of 5 °C/min; Table S1: The feed ratios of samples and the results of the swelling test.
