# Supplementary material for: Fabricating High Strength Bio-Based Dynamic Networks from Epoxidized Soybean Oil and Poly(Butylene Adipate-co-Terephthalate)
Source: Polymers (Basel). 2024 Aug 11;16(16):2280. doi: 10.3390/polym16162280 (PMC11359266; doi:10.3390/polym16162280)
Supplement: Supplementary file 1 [file polymers-16-02280-s001.zip › polymers-3135973-supplementary.pdf]

Supporting Information

Fabricating High Strength Bio-based Dynamic Networks from Epoxidized Soybean Oil and Poly(butylene adipate-*co*-terephthalate)

Bin Xu, Zhong-Ming Xia, Rui Zhan and Ke-Ke Yang *

The Collaborative Innovation Center for Eco-Friendly and Fire-Safety Polymeric Materials (MoE), National Engineering Laboratory of Eco-Friendly Polymeric Materials (Sichuan), College of Chemistry, Sichuan University, Chengdu 610064, China; xvbin1028@126.com (B.X.); 15928876074@163.com (Z.-M.X.); m19942331358@163.com (R.Z.)

* Correspondence: kkyangscu@126.com

Supporting Figures:

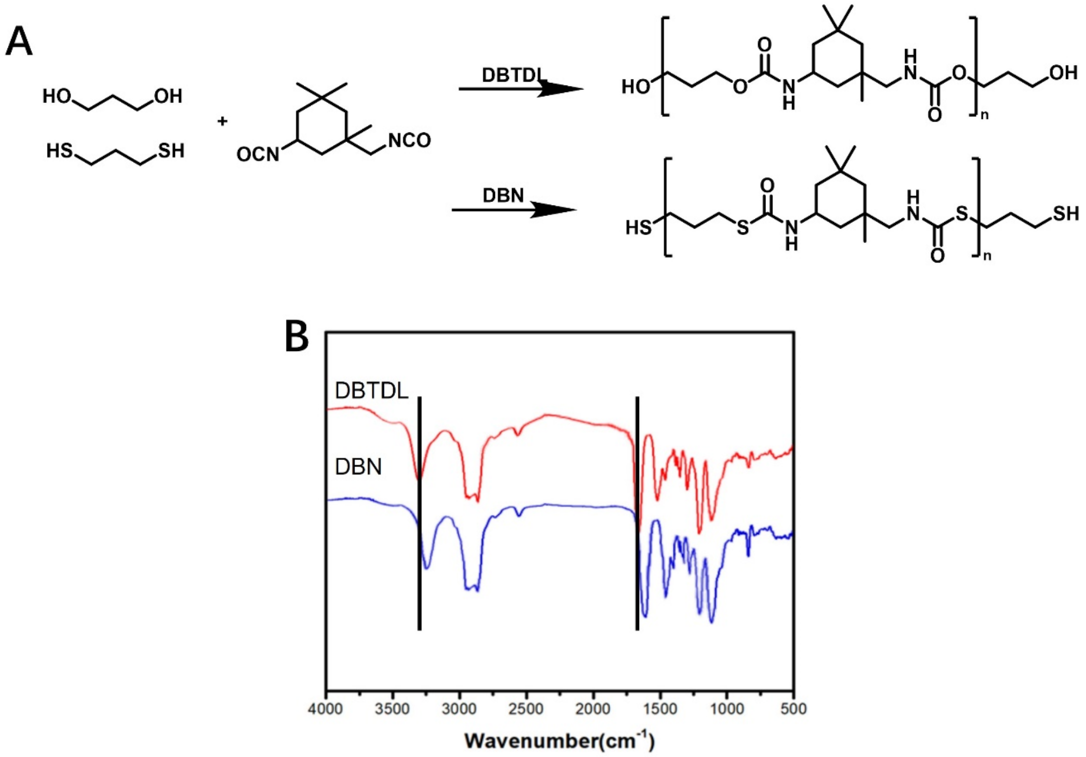

Figure S1 (A) The reaction of IPDI with a mixture of model molecules1,3-Propanedithiol (PDT) and 1,3-Propanediol (PDO) under different catalysts; (B) FT-IR spectra of resultant products

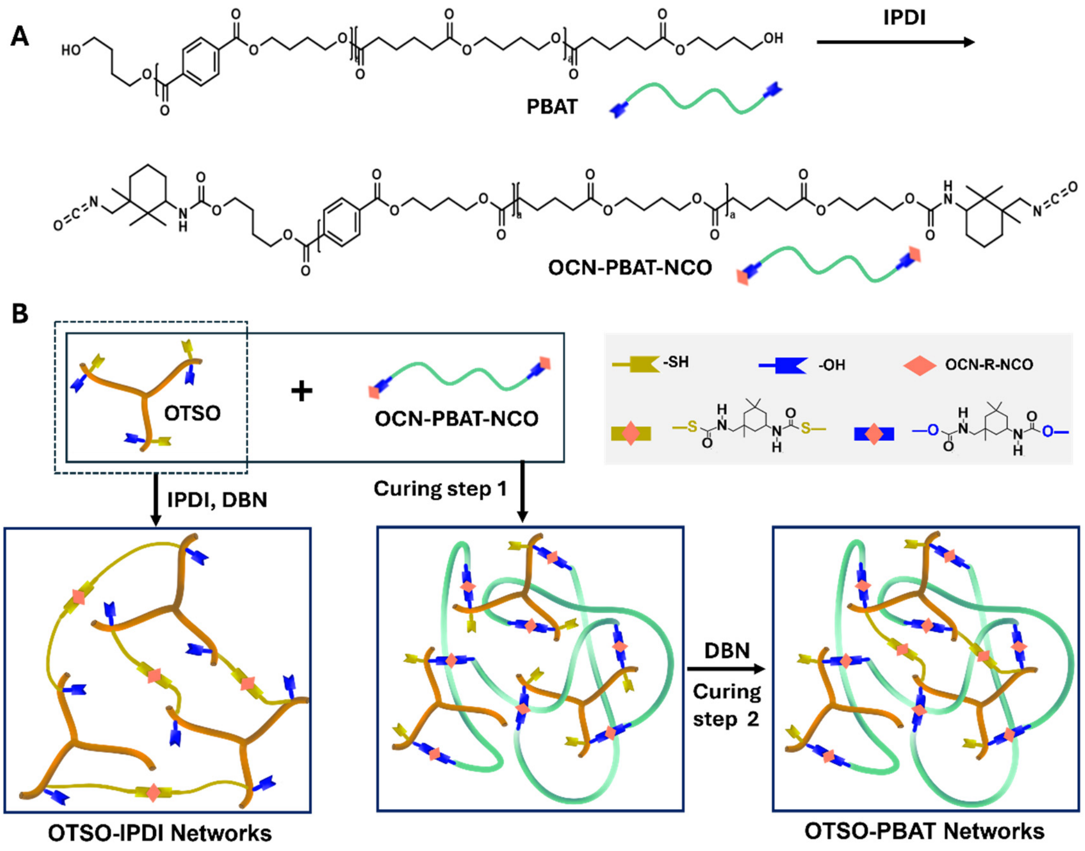

Figure S2 Synthetic strategy of OTSO-IPDI and OTSO-PBAT Networks.

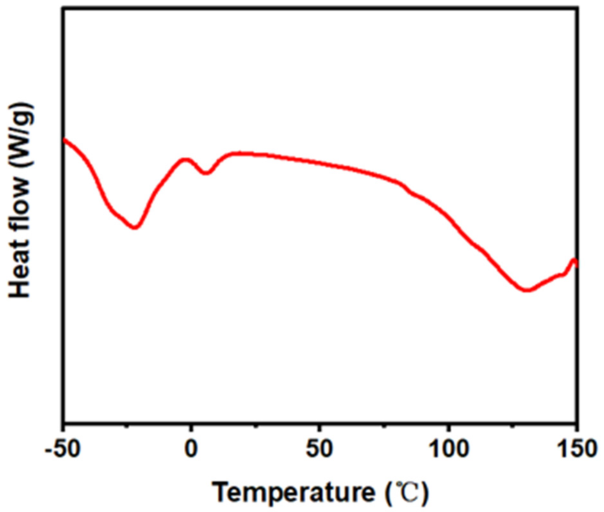

Figure S3 DSC curve of OTSO, NCO-terminated PBAT and IPDI within a temperature range of -50 to 150 °C at a heating rate of 5 °C/min

Table S1 The feed ratios of samples and the results of the swelling test.

Sample	m_{OTSO} (g)	m_{PBAT} (g)	m_{IPDI} (g)	G (%)	S (%)
$OTSO_{100}$-$PBAT_{70}$	0.8	3.5	0.3579	90.1±3.6	869±7
$OTSO_{100}$-$PBAT_{80}$	0.8	4	0.3868	89.0±1.7	910±21
$OTSO_{100}$-$PBAT_{90}$	0.8	4.5	0.4157	87.1±0.4	1070±22
$OTSO_{100}$-$PBAT_{100}$	0.8	5	0.4446	84.2±2.0	1135±17
$OTSO_{100}$-$IPDI_{100}$	0.8	0	0.1545	96.3±0.8	76±14
